# Core Genome Multilocus Sequence Typing and Antibiotic Susceptibility Prediction from Whole-Genome Sequence Data of Multidrug-Resistant Pseudomonas aeruginosa Isolates

**DOI:** 10.1128/spectrum.03920-22

**Published:** 2022-11-09

**Authors:** Scott A. Cunningham, Allison R. Eberly, Stephan Beisken, Andreas E. Posch, Audrey N. Schuetz, Robin Patel

**Affiliations:** a Division of Clinical Microbiology, Department of Laboratory Medicine and Pathology, Mayo Clinic, Rochester, Minnesota, USA; b Ares Genetics GmbH, Vienna, Austria; c Division of Public Health, Infectious Diseases, and Occupational Medicine, Mayo Clinic, Rochester, Minnesota, USA; Houston Methodist Hospital

**Keywords:** core genome multilocus sequence typing, *Psuedomonas aeruginosa*, pulse field gel electrophoresis, whole-genome sequencing

## Abstract

Over the past decade, whole-genome sequencing (WGS) has overtaken traditional bacterial typing methods for studies of genetic relatedness. Further, WGS data generated during epidemiologic studies can be used in other clinically relevant bioinformatic applications, such as antibiotic resistance prediction. Using commercially available software tools, the relatedness of 38 clinical isolates of multidrug-resistant Pseudomonas aeruginosa was defined by two core genome multilocus sequence typing (cgMLST) methods, and the WGS data of each isolate was analyzed to predict antibiotic susceptibility to nine antibacterial agents. The WGS typing and resistance prediction data were compared with pulsed-field gel electrophoresis (PFGE) and phenotypic antibiotic susceptibility results, respectively. Simpson’s Diversity Index and adjusted Wallace pairwise assessments of the three typing methods showed nearly identical discriminatory power. Antibiotic resistance prediction using a trained analytical pipeline examined 342 bacterial-drug combinations with an overall categorical agreement of 92.4% and very major, major, and minor error rates of 3.6, 4.1, and 4.1%, respectively.

**IMPORTANCE** Multidrug-resistant Pseudomonas aeruginosa isolates are a serious public health concern due to their resistance to nearly all or all of the available antibiotics, including carbapenems. Utilizing molecular approaches in conjunction with antibiotic susceptibility prediction software warrants investigation for use in the clinical laboratory workflow. These molecular tools coupled with antibiotic resistance prediction tools offer the opportunity to overcome the extended turnaround time and technical challenges of phenotypic susceptibility testing.

## INTRODUCTION

Pseudomonas aeruginosa is a Gram-negative bacterium that can thrive in a diverse range of environments due to tolerance to various growth conditions and temperatures (1). These traits lend to the classification of P. aeruginosa as an opportunistic pathogen that is frequently associated with hospital-acquired infections, most notably in burn units and in intensive care units. P. aeruginosa is also a pathogen in patients with cystic fibrosis. The species has a large and fluid genome and can possess a diverse array of resistance mechanisms (2, 3). Over the past 5 years, both the World Health Organization (WHO) and the United States Centers for Disease Control and Prevention (CDC) have classified P. aeruginosa as a major threat out of concern for the emergence of both multidrug- and extended-drug-resistant clinical isolates (4, 5). Unfortunately, there is emerging evidence that the global severe acute respiratory syndrome coronavirus 2 (SARS-CoV-2) pandemic may be accelerating antibiotic selective pressure in this species (6).

Medical microbiologists have used a variety of tools to determine the relatedness of P. aeruginosa isolates in suspected outbreaks. Early studies using phenotypic traits such as pyocin production, serologic studies, and antibiograms eventually gave way to pulsed-field gel electrophoresis (PFGE) (7). Alternate molecular methods have been proposed and evaluated over the past two decades, including multilocus sequence typing (MLST), multiple locus variable number tandem-repeat analysis (MLVA), microarrays, and mass spectrometry; however, PFGE remained the most discriminatory method (8–14). Clinical microbiology laboratory access to benchtop next-generation sequencing (NGS) devices and easy-to-use bioinformatic tools allowing for bacterial whole-genome sequencing (WGS) and high-resolution genotyping comparing thousands of alleles has disrupted PFGE as the gold standard typing method.

Juxtaposed to bacterial typing, antimicrobial susceptibility testing (AST) has remained largely unchanged. Conventional AST is highly standardized and based on phenotypic observations of growth inhibition in broth or on solid media. Conventional AST turnaround may take as long as 3 to 4 days from primary isolation. Further, the laboratory can encounter isolates that fail to grow on the acceptable AST media, leaving care providers without laboratory data to guide antibiotic selection. Establishment of a molecular approach has the potential to overcome both extended AST turnaround time and circumvent failure of isolate growth for AST.

Our group has established species-independent standardized clinical laboratory workflows for extraction, NGS library preparation, and WGS and applied the data for both molecular typing and antibiotic resistance prediction in a way that laboratory work and preliminary bioinformatics tasks are performed by bench-level medical laboratory science staff (15–17). In this work, comparative data on two core genome multilocus sequence typing (cgMLST) methods and PFGE are presented, and the performance of a commercial resistance prediction pipeline compared to agar dilution AST are evaluated using a collection of 38 drug-resistant P. aeruginosa isolates.

## RESULTS

### PFGE typing.

Two clonal groups consisting of 18 (P6) and 4 (P2) isolates, respectively, were identified, the remaining 16 isolates had unique banding patterns and were assigned independent nomenclature (Table 4).

### cgMLST typing.

**FIG 1:**
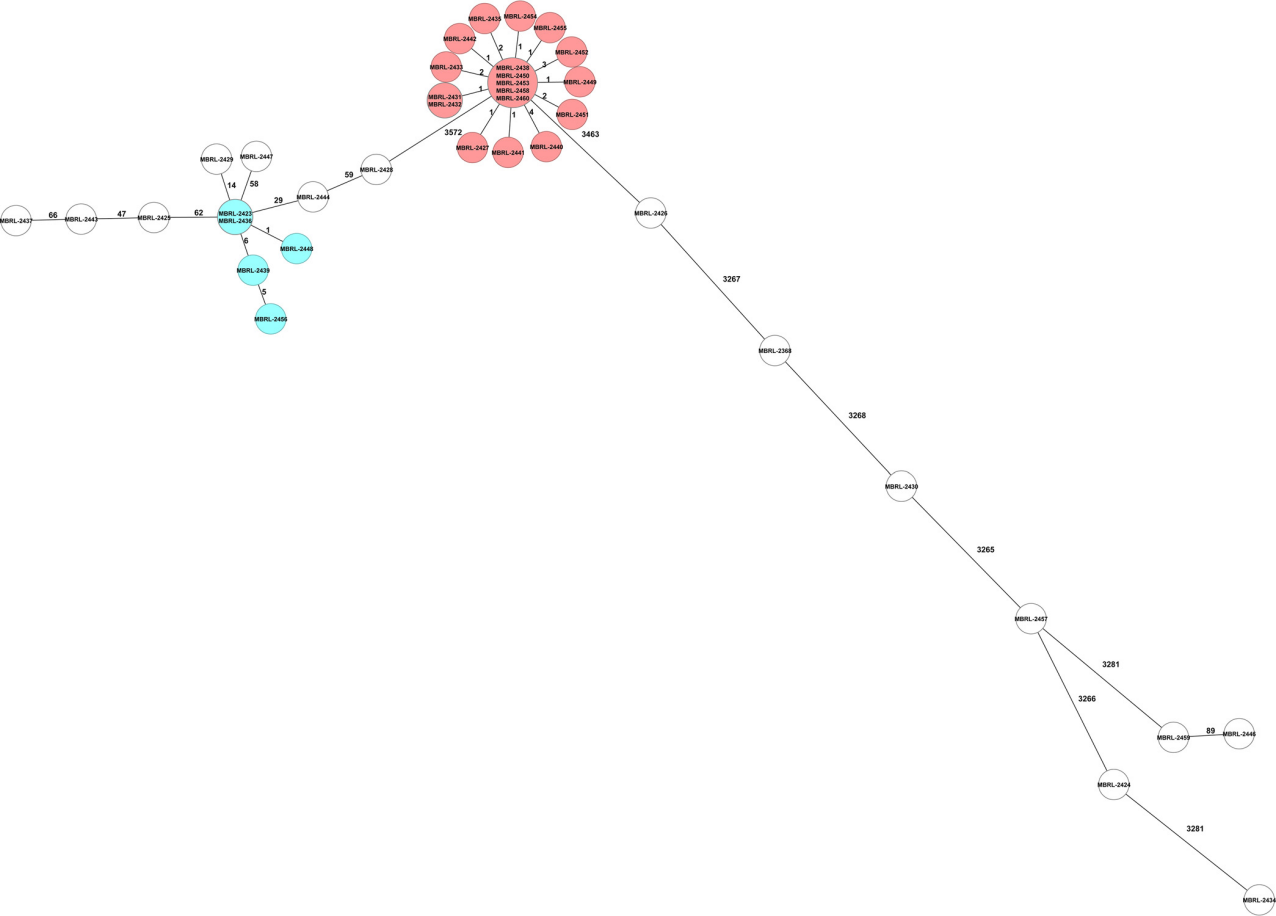
Mayo core genome multilocus sequence (cgMLST) typing minimum spanning tree.

**(i) Mayo cgMLST.** Using the relatedness cutoff of six or fewer allelic differences, the Mayo cgMLST method identified two clonal clusters of isolates consisting of 18 (M6) and 5 (M2) isolates. The 15 remaining isolates were considered unrelated and assigned independent nomenclature (Fig. 1, Table 4).

**FIG 2:**
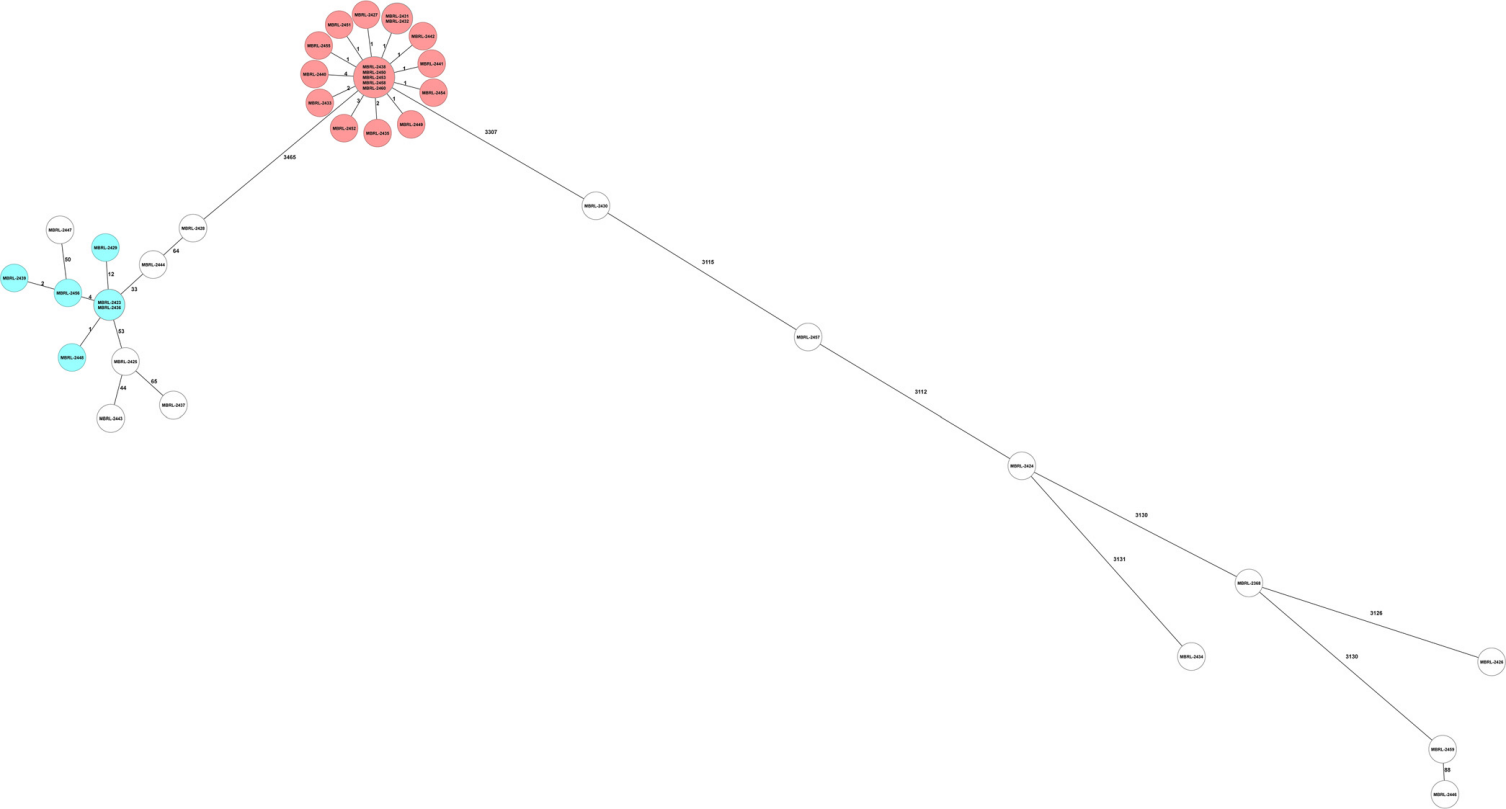
SeqSphere+ core genome multilocus sequence typing (cgMSLT) minimum spanning tree.

**(ii) SeqSphere+ cgMLST.** The SeqSphere+ cgMLST method provides a proposed standardized nomenclature (complex type). The assigned complex types were utilized to define clonal clusters for this analysis. This method identified two clonal clusters of isolates consisting of 18 (2,802) and 6 (2,798) isolates; the remaining 14 isolates were considered unrelated and assigned unique complex type identifiers (Fig. 2, Table 4).

### Statistical comparison of typing methods.

Comparison of the three methods by Simpson’s Diversity Index revealed overlapping 95% confidence intervals for all three methods, suggesting that all methods had similar discriminatory power (Table 1). Pairwise comparison of the three methods by the adjusted Wallace showed minor differences between the three methods. Adjusted Wallace_SeqSphere+ cgMLST→Mayo cgMLST_ = 1.00, and adjusted Wallace_Mayo cgMLST→SeqSphere+ cgMLST_ = 0.954 (95% CI = 0.903 to 1.000); adjusted Wallace_SeqSphere+ cgMLST→PFGE_ = 1.00, and adjusted Wallace_PFGE→SeqSphere+ cgMLST_ = 0.924 (95% CI = 0.874 to 0.973); adjusted Wallace_Mayo cgMLST→PFGE_ = 1.00m and adjusted Wallace_PFGE→Mayo cgMLST_ = 0.968 (95% CI = 0.931 to 1.000) (Table 2).

**TABLE 1:** Typing method comparison by Simpson’s Diversity Index^*a*^

Test name	No. of partitions	Simpson’s diversity index	Confidence interval (95%)
Pulsed-field gel electrophoresis	17	0.768	0.630 to 0.906
SeqSphere+ cgMLST	15	0.760	0.625 to 0.894
Mayo cgMLST	17	0.768	0.630 to 0.906

*^a^*cgMLST, core genome multilocus sequence typing.

**TABLE 2:** Typing method comparison by adjusted Wallace tests^*a*^

Test name	Pulsed-field gel electrophoresis	SeqSphere+ cgMLST	Mayo cgMLST
Pulsed-field gel electrophoresis		1.000 (1.000 to 1.000)	0.968 (0.930 to 1.000)
SeqSphere+ cgMLST	0.954 (0.903 to 1.000)		0.954 (0.903 to 1.000)
Mayo cgMLST	0.968 (0.930 to 1.000)	1.000 (1.000 to 1.000)	

*^a^* The values are shown as the adjusted Wallace test result (95% confidence interval). cgMLST, core genome multilocus sequence typing.

### Agar dilution.

Resistance rates were high and spanned several drug classes. The highest phenotypic resistance rates were with meropenem (84%) and the two fluoroquinolone antibiotics, ciprofloxacin and levofloxacin (84%). Gentamicin resistance (84%) was slightly higher than tobramycin resistance (82%). Aztreonam, ceftazidime, and piperacillin-tazobactam resistance were all noted as 79%, and the cefepime resistance rate was 76%.

### ARESdb analysis.

In total, there were 342 results for the 9 antibiotics surveyed. Overall categorical agreement (CA) between agar dilution (AD) and ARESdb genotypic prediction was 92.4% (316 of 342) with a very major error rate (VME) of 3.6% (10 of 279), a major error rate (ME) of 4.1% (2 of 49), and a minor error rate (mE) of 4.1% (14 of 342).

The β-lactam class CA varied within the class. Piperacillin-tazobactam had the lowest CA (84.2%, 32 of 38) with a VME of 10% (3 of 30) and no ME. CA for ceftazidime was 86.8% (33 of 38) with a 6.7% (2 of 30) VME and no ME. CA for aztreonam was 89.5% (34 of 38) with a VME of 3.3% (1 of 30) and no ME. Only cefepime and meropenem had CA values >90% (92.1% [35 of 38] and 97.4% [37 of 38], respectively). Cefepime did not have any VMEs or MEs; inaccuracies were all attributable to the lack of an intermediate prediction. Meropenem had a 3.1% (1 of 32) VME rate attributable to a single isolate.

Gentamicin CA was 89.5% (34 of 38) with VME and ME rates of 6.3% (2 of 32) and 20% (1 of 5), respectively. Tobramycin had a slightly better CA of 94.7% (36 of 38), with VME and ME rates of 3.1% (1 of 32) and 16.7% (1 of 6), respectively.

Finally, the fluoroquinolones had excellent CA overall. Ciprofloxacin had perfect CA, and levofloxacin CA was 97.4% (37 of 38). Inaccuracies within the levofloxacin CA were all attributable to the lack of intermediate results by ARESdb.

## DISCUSSION

The application of WGS for investigation of genomic relatedness in epidemiologic studies has replaced PFGE in recent years. The current study compared two cgMLST schemes with a differing number of core genome alleles within the same software (SeqSphere+) against PFGE. Simpson’s Diversity Index 95% CI values were nearly perfectly overlapping, indicating that the three methods evaluated here had similar discriminatory power (Table 1). In pairwise comparisons, the three methods were nearly equivalent (Tables 1 and 2). Any two isolates within a cluster predicted by SeqSphere+ cgMLST scheme would have a 100% chance of falling into the same cluster using the Mayo cgMLST. Conversely, there was a 95.4% chance of a Mayo cgMLST pairing falling into the same SeqSphere+ cluster. It was not possible to determine whether the difference between the two methods was due to the allelic threshold applied in the Mayo cgMLST scheme. A single isolate in cluster SeqSphere+ cgMLST type 2802 was included with a value of 12 allelic differences (Fig. 2). The methods perfectly align if an allelic threshold of ≤6 is applied to the SeqSphere+ cgMLST data set. Any two isolates that fell into a cluster in the SeqSphere+ cgMLST scheme had a 100% chance of falling into the same cluster by PFGE. Any two isolates falling into a cluster in the Mayo cgMLST scheme had a 96.8% chance of falling into the same cluster by PFGE. Conversely, there was a 95.4% chance that cluster pairings identified with PFGE would fall into the same cluster in the SeqSphere+ cgMLST scheme. Similarly, there was a 96.8% chance that a cluster pairing identified by PFGE would fall into the same cluster in the Mayo cgMLST. These data align with the findings of Martak et al. (18). In that study of 65 isolates, the investigators concluded that PFGE could be used “with confidence” in the investigation of localized P. aeruginosa outbreaks. Further, they noted that WGS-based typing was not as affected by recombination events as PFGE (18). In the data set, isolate MBRL-2439 was missing a single band, which excluded it from PFGE group P2 affecting the overall agreement of PFGE with the two cgMLST methods. We hypothesize that the single-band difference may have been due to a recombination event affecting the restriction enzyme cut site but not comparison at the allele level (19).

*In silico* resistance prediction in P. aeruginosa using the ARESdb pipeline appears to be promising. Strong confidence in resistance prediction for the fluoroquinolones (ciprofloxacin and levofloxacin) was found, with no VMEs or MEs noted and CA values >95%. Meropenem had a high CA (97.4%, 37 of 38) but had a single false susceptible call, resulting in a VME of 3.1% (1 of 32). Cefepime prediction (CA = 92.1%, 35 of 38) was also acceptable, with no VMEs or MEs. The overall CA was brought down by mEs, which is a limitation of the ARESdb prediction pipeline. Finally, tobramycin prediction (CA = 94.7%, 36 of 38) was acceptable, but a high ME rate was observed, with one false resistant call (16.7%, 1 of 6) (Table 3).

**TABLE 3:** ARESdb prediction statistics^*a*^

Compound	CA	VME	ME	mE
Aztreonam	89.5% (34 of 38)	3.3% (1 of 30)	0.0%	7.9% (3 of 38)
Cefepime	92.1% (35 of 38)	0.0%	0.0%	7.9% (3 of 38)
Ceftazidime	86.8% (33 of 38)	6.7% (2 of 30)	0.0%	7.9% (3 of 38)
Ciprofloxacin	100.0% (38 of 38)	0.0%	0.0%	0.0%
Gentamicin	89.5% (34 of 38)	6.3% (2 of 38)	20.0% (1 of 5)	2.6% (1 of 38)
Levofloxacin	97.4% (37 of 38)	0.0%	0.0%	2.6% (1 of 38)
Meropenem	97.4% (37 of 38)	3.1% (1 of 32)	0.0%	0.0%
Piperacillin-tazobactam	84.2% (32 of 38)	10.0% (3 of 30)	0.0%	7.9% (3 of 38)
Tobramycin	94.7% (36 of 38)	3.1% (1 of 32)	16.7% (1 of 6)	0.0%
				
Overall	92.4% (316 of 342)	3.7% (10 of 269)	4.3% (2 of 47)	4.1% (14 of 342)

*^a^*CA, categorical agreement; VME, very major error; ME, major error; mE, minor error.

The remaining compound predictions were all <90% CA and encompassed the drug classes of β-lactams and aminoglycosides. Aztreonam had a CA of 89.5% and a single false susceptible prediction (VME = 3.3%, 1 of 30), with a mE rate of 7.9% (3 of 38) lowering overall CA. Ceftazidime prediction experienced two false susceptible calls (VME = 6.7%, 2 of 30), piperacillin-tazobactam prediction experienced three false susceptible calls (VME = 10%, 3 of 30), and gentamicin prediction experience two false susceptible calls (Table 3).

It has been noted in several studies that genotypic prediction of resistance in P. aeruginosa is complex, especially for the β-lactams and aminoglycosides. P. aeruginosa is known to harbor a drug-inducible AmpC β-lactamase Pseudomonas-derived cephalosporinase (PDC) leading to natural resistance to the penicillins and first and second generation cephalosporins (20, 21). Some mutations in PDCs extend the spectrum of the natural activity of these enzymes to include piperacillin-tazobactam, the antipseudomonal cephalosporins, and the monobactam aztreonam (20). Further complicating this landscape, overexpression of PDCs, modifications of OprD, upregulation of the efflux pump system (*mex*), interplay with other β-lactamases such as the group D oxacillinases, and the mucoid phenotype of this species can affect β-lactam MICs (20, 22–25). Similarly, *in silico* prediction of aminoglycoside resistance is complex and has also been demonstrated to be multifactorial. P. aeruginosa can inactivate aminoglycosides with a number of aminoglycoside-modifying enzymes that have various activities against the different agents in this class. Additionally, this species can possess 16S rRNA gene methyl transferases, which can render aminoglycosides inactive (26–28). Finally, the efflux pump system MexXY-OprM has been demonstrated to contribute to aminoglycoside resistance (29).

We attempted to use the National Center for Biotechnology (NCBI) antibiotic resistance gene identifying tool AMRFinderPlus, which was recently added into SeqSphere+ (30). AMRFinderPlus has several species-specific pathways included that allow for more precise predictions. Currently AMRFinderPlus does not have a P. aeruginosa resistance prediction pathway. Users are still able to analyze assembly FASTA files without defining the species queried, and gene-call results with color-coded confidence scores are provided. The end user must have broad knowledge regarding single-gene and gene-environment interplay mechanisms that confer resistance to manually correlate identified antimicrobial resistance genes with resistance prediction. In our hands, this task was difficult and time consuming, and the results had poor CA (data not shown).

Cortes-Lara et al. employed a laboratory-developed *in silico* prediction tool and conducted a similar resistance prediction study with a larger collection of P. aeruginosa isolates (31). The laboratory-developed tool utilizes a logic-based scoring system that takes into account the presence/absence of genes and point mutations associated with increased resistance or susceptibility. The team compared European Committee on Antimicrobial Susceptibility Testing (EUCAST)-interpreted AST data for five antibiotics to predictions made with the analytical tool. The reported sensitivities were 86.5% (meropenem), 95.5% (ciprofloxacin), 97.7% (ceftazidime), and 100% (ceftolozane-tazobactam and tobramycin). These data suggest an advantage of built-in “logic” to assist in making resistance predictions for this species (31). In another recent study using a machine learning approach, investigators combined a DNA antimicrobial resistance (AMR) identification tool with and without transcriptome sequencing to enable parallel expression analysis. They noted improved performance in prediction of antibiotic resistance when expression analysis was included (32). Future studies will require the development of optimized *in silico* resistance tools and databases and assessment of these tools in a multicenter approach. Limitations of this study include the use of a small number of isolates with moderate diversity and a limited antibiotic prediction panel, which does not include some of the new β-lactam inhibitor combinations that can inhibit PDCs.

Resistance prediction using WGS data is an emerging arena in clinical microbiology. WGS resistance prediction from bacterial isolates has the potential to be a bedrock to enable future resistance prediction from data derived from culture-independent molecular techniques, such as shotgun metagenomics. Any *in silico* resistance prediction tool used for P. aeruginosa will require a robust database and a degree of logic or artificial intelligence to account for the diverse genetic pathways that can lead to antibiotic resistance.

## MATERIALS AND METHODS

### Bacterial isolates.

The study used 38 isolates submitted to the Mayo Clinic Clinical Bacteriology Laboratory for PFGE analysis. Isolates were revived from cryopreservation by two serial passages onto trypticase soy agar with 5% sheep blood (Becton Dickinson, Sparks, MD) and incubated in an ambient atmosphere at 35°C. The second serial passage was used for both conventional AST and molecular methods.

### Pulsed-field gel electrophoresis (PFGE).

PFGE was performed on all 38 isolates by visually capturing and comparing banding profiles. Briefly, PFGE was performed by creating cell suspensions for all isolates in Tris-EDTA buffer to an optical density of 45 to 55% transmittance at 590-nm turbidity (Dade Behring, Deerfield, IL). Normalized cells were embedded in sample agarose plugs and digested in a lysis buffer containing 200 mg/μL lysozyme and 0.05% sarkosyl for 4 h at 35°C. At the conclusion of incubation, lysis buffer was neutralized and washed away. Each plug was transferred to a microcentrifuge tube containing restriction buffer H and 30 units of XbaI enzyme (Sigma-Aldrich, St. Louis, MO) and incubated for an additional 4 h at 35°C. PFGE was performed on a CHEF Mapper XA system (Bio-Rad, Hercules, CA). PFGE gels were stained with ethidium bromide and visualized on a GelDoc system (Bio-Rad). Comparison of banding patterns was manually performed.

### Whole-genome sequencing (WGS).

DNA was extracted and purified using the Quick-DNA fungal/bacterial miniprep kit (Zymo Research Corp.) in 200 μL of the kit-provided elution buffer. DNA was measured and normalized to 0.2 ng/μL using the QuantiFluor ONE dsDNA system with a Quantus fluorometer (Promega, Madison, WI). NGS libraries were prepared from the normalized DNA with Nextera XT (Illumina, San Diego, CA) and dual-indexed. Sequencing was performed on a MiSeq benchtop sequencer (Illumina) utilizing a V2 500 cycle paired-end kit with a maximum pooling targeting 200× mean genome coverage. The reads were processed for adapter and index cleaning using MiSeq reporter software in real time.

### cgMLST analysis.

Adapter- and index-clipped read files were imported into SeqSphere+ (Ridom, Münster, Germany) software version 7.2.6. SKESA *de novo* assembly and two cgMLST analyses were exercised within the software suite following default settings. An *ad hoc* cgMLST method developed at Mayo Clinic in 2019 that analyzes 4,041 alleles based on the PAO1 reference genome (NC_002516.2) was run in parallel and compared to the cgMLST method of Tönnies et al., which employs the same reference genome (albeit with fewer alleles) but offers a proposed standard cgMLST nomenclature and has been incorporated within the SeqSphere+ software (33). Minimum spanning trees were generated using the comparison data from both methods. The following laboratory-validated allelic thresholds of relatedness were applied to the Mayo cgMLST method: ≤6 allelic differences, related; 7 to 100 allelic differences, possibly related; and >101 allelic differences, unrelated.

### Statistical comparison of typing methods.

PFGE and Mayo cgMLST groupings were assigned arbitrary values in the absence of formalized nomenclature (Table 4). Typing data were uploaded for analysis into the Comparing Partitions tool (www.comparingpartitions.info). The three methods were analyzed collectively with Simpson’s Diversity Index and pairwise with the adjusted Wallace test (34, 35).

**TABLE 4:** PFGE and cgMLST typing data^*a*^

Isolate ID	SeqSphere+ cgMLST complex type	Mayo cgMLST complex type	PFGE grouping
MBRL2368	2814	M1	P1
MBRL2423	2798	M2	P2
MBRL2424	2799	M3	P3
MBRL2425	2800	M4	P4
MBRL2426	2801	M5	P5
MBRL2427	2802	M6	P6
MBRL2428	2809	M7	P7
MBRL2429	2798	M8	P2
MBRL2430	2804	M9	P9
MBRL2431	2802	M6	P6
MBRL2432	2802	M6	P6
MBRL2433	2802	M6	P6
MBRL2434	2805	M10	P10
MBRL2435	2802	M6	P6
MBRL2436	2798	M2	P2
MBRL2437	2806	M11	P11
MBRL2438	2802	M6	P6
MBRL2439	2798	M2	P12
MBRL2440	2802	M6	P6
MBRL2441	2802	M6	P6
MBRL2442	2802	M6	P6
MBRL2443	2807	M12	P13
MBRL2444	2808	M13	P14
MBRL2446	2809	M14	P15
MBRL2447	2810	M15	P16
MBRL2448	2798	M2	P2
MBRL2449	2802	M6	P6
MBRL2450	2802	M6	P6
MBRL2451	2802	M6	P6
MBRL2452	2802	M6	P6
MBRL2453	2802	M6	P6
MBRL2454	2802	M6	P6
MBRL2455	2802	M6	P6
MBRL2456	2798	M2	P2
MBRL2457	2811	M16	P18
MBRL2458	2802	M6	P6
MBRL2459	2812	M17	P19
MBRL2460	2802	M6	P6

*^a^*cgMLST, core genome multilocus sequence typing; PFGE, pulsed-field gel electrophoresis.

### Agar dilution.

Phenotypic AST was performed using AD following quality control and breakpoints using the Clinical and Laboratory Standards Institute M100 32nd edition document (36).

### ARESdb analysis.

Adapter- and index-clipped read files were uploaded into the ARESdb platform, release 2020-04 (Ares Genetics GmbH, Vienna, Austria) for genomic prediction of antimicrobial susceptibility. The platform used susceptibility/resistance (S/R) stacked classification models trained per species-compound pair on the AMR reference database ARESdb (37). Nine compound-specific stacked classification models were used for genomic prediction of susceptibility based on the uploaded data. Intermediate phenotypes were treated as minor errors, because the ARESdb platform did not predict the intermediate interpretive category. Very major error (VME), major error (ME), and minor error (mE) rates were calculated following CLSI M52 guidelines (38).

### Data availability.

Sequences from isolates in this study are available in the National Center for Biotechnology (NCBI) Sequence Read Archive (SRA) under Accession PRJNA855929.

## References

[1] Diggle SP, Whiteley M. 2020. Microbe profile: *Pseudomonas aeruginosa*: opportunistic pathogen and lab rat. Microbiology 166:30–33. doi:10.1099/mic.0.000860. 31597590 PMC7273324

[2] Botelho J, Grosso F, Peixe L. 2019. Antibiotic resistance in *Pseudomonas aeruginosa*—mechanisms, epidemiology and evolution. Drug Resist Updat 44:100640. doi:10.1016/j.drup.2019.07.002. 31492517

[3] Pang Z, Raudonis R, Glick BR, Lin TJ, Cheng Z. 2019. Antibiotic resistance in *Pseudomonas aeruginosa*: mechanisms and alternative therapeutic strategies. Biotechnol Adv 37:177–192. doi:10.1016/j.biotechadv.2018.11.013. 30500353

[4] Centers for Disease Control and Prevention National Center for Emerging and Zoonotic Infectious Diseases (NCEZID), Division of Healthcare Quality Promotion (DHQP). 2021. Antibiotic Resistance Threats in the United States, 2019. https://www.cdc.gov/drugresistance/biggest-threats.html.

[5] World Health Organization. 2017. WHO publishes list of bacteria for which new antibiotics are urgently needed. https://www.who.int/news/item/27-02-2017-who-publishes-list-of-bacteria-for-which-new-antibiotics-are-urgently-needed.

[6] Kariyawasam RM, Julien DA, Jelinski DC, Larose SL, Rennert-May E, Conly JM, Dingle TC, Chen JZ, Tyrrell GJ, Ronksley PE, Barkema HW. 2022. Antimicrobial resistance (AMR) in COVID-19 patients: a systematic review and meta-analysis (November 2019–June 2021). Antimicrob Resist Infect Control 11:45. doi:10.1186/s13756-022-01085-z. 35255988 PMC8899460

[7] Bobo RA, Newton EJ, Jones LF, Farmer LH, Farmer JJ, III. 1973. Nursery outbreak of *Pseudomonas aeruginosa*: epidemiological conclusions from five different typing methods. Appl Microbiol 25:414–420. doi:10.1128/am.25.3.414-420.1973. 4633428 PMC380820

[8] Ballarini A, Scalet G, Kos M, Cramer N, Wiehlmann L, Jousson O. 2012. Molecular typing and epidemiological investigation of clinical populations of *Pseudomonas aeruginosa* using an oligonucleotide-microarray. BMC Microbiol 12:152. doi:10.1186/1471-2180-12-152. 22840192 PMC3431270

[9] Fleurbaaij F, Kraakman ME, Claas EC, Knetsch CW, van Leeuwen HC, van der Burgt YE, Veldkamp KE, Vos MC, Goessens W, Mertens BJ, Kuijper EJ, Hensbergen PJ, Nicolardi S. 2016. Typing *Pseudomonas aeruginosa* isolates with ultrahigh resolution MALDI-FTICR mass spectrometry. Anal Chem 88:5996–6003. doi:10.1021/acs.analchem.6b01037. 27123572

[10] Jarych D, Augustynowicz-Kopec E, Iwanska A, Parniewski P, Majchrzak M. 2021. Molecular analysis of *Pseudomonas aeruginosa* strains isolated from cystic fibrosis patients. Sci Rep 11:15460. doi:10.1038/s41598-021-95034-2. 34326452 PMC8322141

[11] Johansson E, Welinder-Olsson C, Gilljam M, Pourcel C, Lindblad A. 2015. Genotyping of *Pseudomonas aeruginosa* reveals high diversity, stability over time and good outcome of eradication. J Cyst Fibros 14:353–360. doi:10.1016/j.jcf.2014.09.016. 25458462

[12] Johnson JK, Arduino SM, Stine OC, Johnson JA, Harris AD. 2007. Multilocus sequence typing compared to pulsed-field gel electrophoresis for molecular typing of *Pseudomonas aeruginosa*. J Clin Microbiol 45:3707–3712. doi:10.1128/JCM.00560-07. 17881548 PMC2168506

[13] Renders N, van Belkum A, Barth A, Goessens W, Mouton J, Verbrugh H. 1996. Typing of *Pseudomonas aeruginosa* strains from patients with cystic fibrosis: phenotyping versus genotyping. Clin Microbiol Infect 1:261–265. doi:10.1016/S1198-743X(15)60285-3. 11866776

[14] Waters V, Zlosnik JE, Yau YC, Speert DP, Aaron SD, Guttman DS. 2012. Comparison of three typing methods for *Pseudomonas aeruginosa* isolates from patients with cystic fibrosis. Eur J Clin Microbiol Infect Dis 31:3341–3350. doi:10.1007/s10096-012-1701-z. 22843295

[15] Banerjee R, Cunningham SA, Beisken S, Posch AE, Johnston B, Johnson JR, Patel R. 2021. Core genome multilocus sequence typing and prediction of antimicrobial susceptibility using whole-genome sequences of *Escherichia coli* bloodstream infection isolates. Antimicrob Agents Chemother 65:e0113921. doi:10.1128/AAC.01139-21. 34424049 PMC8522740

[16] Cunningham SA, Jeraldo PR, Schuetz AN, Heitman AA, Patel R. 2020. *Staphylococcus aureus* whole genome sequence-based susceptibility and resistance prediction using a clinically amenable workflow. Diagn Microbiol Infect Dis 97:115060. doi:10.1016/j.diagmicrobio.2020.115060. 32417617 PMC8094441

[17] Fida M, Cunningham SA, Murphy MP, Bonomo RA, Hujer KM, Hujer AM, Kreiswirth BN, Chia N, Jeraldo PR, Nelson H, Zinsmaster NM, Toraskar N, Chang W, Patel R, Antibacterial Resistance Leadership Group. 2020. Core genome MLST and resistome analysis of *Klebsiella pneumoniae* using a clinically amenable workflow. Diagn Microbiol Infect Dis 97:114996. doi:10.1016/j.diagmicrobio.2020.114996. 32098688 PMC7422488

[18] Martak D, Meunier A, Sauget M, Cholley P, Thouverez M, Bertrand X, Valot B, Hocquet D. 2020. Comparison of pulsed-field gel electrophoresis and whole-genome-sequencing-based typing confirms the accuracy of pulsed-field gel electrophoresis for the investigation of local *Pseudomonas aeruginosa* outbreaks. J Hosp Infect 105:643–647. doi:10.1016/j.jhin.2020.06.013. 32585172

[19] Tenover FC, Arbeit RD, Goering RV, Mickelsen PA, Murray BE, Persing DH, Swaminathan B. 1995. Interpreting chromosomal DNA restriction patterns produced by pulsed-field gel electrophoresis: criteria for bacterial strain typing. J Clin Microbiol 33:2233–2239. doi:10.1128/jcm.33.9.2233-2239.1995. 7494007 PMC228385

[20] Berrazeg M, Jeannot K, Ntsogo Enguene VY, Broutin I, Loeffert S, Fournier D, Plesiat P. 2015. Mutations in beta-Lactamase AmpC increase resistance of *Pseudomonas aeruginosa* isolates to antipseudomonal cephalosporins. Antimicrob Agents Chemother 59:6248–6255. doi:10.1128/AAC.00825-15. 26248364 PMC4576058

[21] Jacoby GA. 2009. AmpC beta-lactamases. Clin Microbiol Rev 22:161–182. doi:10.1128/CMR.00036-08. 19136439 PMC2620637

[22] Hao M, Ma W, Dong X, Li X, Cheng F, Wang Y. 2021. Comparative genome analysis of multidrug-resistant *Pseudomonas aeruginosa* JNQH-PA57, a clinically isolated mucoid strain with comprehensive carbapenem resistance mechanisms. BMC Microbiol 21:133. doi:10.1186/s12866-021-02203-4. 33932986 PMC8088628

[23] Jorth P, McLean K, Ratjen A, Secor PR, Bautista GE, Ravishankar S, Rezayat A, Garudathri J, Harrison JJ, Harwood RA, Penewit K, Waalkes A, Singh PK, Salipante SJ. 2017. Evolved aztreonam resistance is multifactorial and can produce hypervirulence in *Pseudomonas aeruginosa*. mBio 8:e00517-17. doi:10.1128/mBio.00517-17. 29089424 PMC5666152

[24] Quale J, Bratu S, Gupta J, Landman D. 2006. Interplay of efflux system, ampC, and oprD expression in carbapenem resistance of *Pseudomonas aeruginosa* clinical isolates. Antimicrob Agents Chemother 50:1633–1641. doi:10.1128/AAC.50.5.1633-1641.2006. 16641429 PMC1472219

[25] Rodriguez-Martinez JM, Poirel L, Nordmann P. 2009. Extended-spectrum cephalosporinases in *Pseudomonas aeruginosa*. Antimicrob Agents Chemother 53:1766–1771. doi:10.1128/AAC.01410-08. 19258272 PMC2681535

[26] Doi Y, Wachino J-I, Arakawa Y. 2016. Aminoglycoside resistance: the emergence of acquired 16S ribosomal RNA methyltransferases. Infect Dis Clin North Am 30:523–537. doi:10.1016/j.idc.2016.02.011. 27208771 PMC4878400

[27] Ramirez MS, Tolmasky ME. 2010. Aminoglycoside modifying enzymes. Drug Resist Updat 13:151–171. doi:10.1016/j.drup.2010.08.003. 20833577 PMC2992599

[28] Shaw KJ, Rather PN, Hare RS, Miller GH. 1993. Molecular genetics of aminoglycoside resistance genes and familial relationships of the aminoglycoside-modifying enzymes. Microbiol Rev 57:138–163. doi:10.1128/mr.57.1.138-163.1993. 8385262 PMC372903

[29] Atassi GS, Scheetz M, Nozick S, Rhodes NJ, Murphy-Belcaster M, Murphy KR, Ozer EA, Hauser AR. 2021. Genomics of aminoglycoside resistance in *Pseudomonas aeruginosa* bloodstream infections at a United States academic hosipital. medRxiv. doi:10.1101/2021.01.15.21249897. PMC10269721 37191517

[30] Feldgarden M, Brover V, Gonzalez-Escalona N, Frye JG, Haendiges J, Haft DH, Hoffmann M, Pettengill JB, Prasad AB, Tillman GE, Tyson GH, Klimke W. 2021. AMRFinderPlus and the Reference Gene Catalog facilitate examination of the genomic links among antimicrobial resistance, stress response, and virulence. Sci Rep 11:12728. doi:10.1038/s41598-021-91456-0. 34135355 PMC8208984

[31] Cortes-Lara S, Barrio-Tofino ED, Lopez-Causape C, Oliver A, GEMARA-SEIMC/REIPI Pseudomonas Study Group 1. 2021. Predicting *Pseudomonas aeruginosa* susceptibility phenotypes from whole genome sequence resistome analysis. Clin Microbiol Infect 27:1631–1637. doi:10.1016/j.cmi.2021.05.011. 34015532

[32] Khaledi A, Weimann A, Schniederjans M, Asgari E, Kuo TH, Oliver A, Cabot G, Kola A, Gastmeier P, Hogardt M, Jonas D, Mofrad MR, Bremges A, McHardy AC, Haussler S. 2020. Predicting antimicrobial resistance in *Pseudomonas aeruginosa* with machine learning-enabled molecular diagnostics. EMBO Mol Med 12:e10264. doi:10.15252/emmm.201910264. 32048461 PMC7059009

[33] Tönnies H, Prior K, Harmsen D, Mellmann A. 2021. Establishment and evaluation of a core genome multilocus sequence typing scheme for whole-genome sequence-based typing of *Pseudomonas aeruginosa*. J Clin Microbiol 59:e01987-20. doi:10.1128/JCM.01987-20. 33328175 PMC8106710

[34] Carrico JA, Silva-Costa C, Melo-Cristino J, Pinto FR, de Lencastre H, Almeida JS, Ramirez M. 2006. Illustration of a common framework for relating multiple typing methods by application to macrolide-resistant *Streptococcus pyogenes*. J Clin Microbiol 44:2524–2532. doi:10.1128/JCM.02536-05. 16825375 PMC1489512

[35] Hunter PR, Gaston MA. 1988. Numerical index of the discriminatory ability of typing systems: an application of Simpson's index of diversity. J Clin Microbiol 26:2465–2466. doi:10.1128/jcm.26.11.2465-2466.1988. 3069867 PMC266921

[36] Clinical Laboratory Standards Institute. 2022. M100 Performance Standards for Antimicrobial Susceptibility Testing. 32nd ed. Clinical Laboratory Standards Institute, Wayne, PA.

[37] Ferreira I, Beisken S, Lueftinger L, Weinmaier T, Klein M, Bacher J, Patel R, von Haeseler A, Posch AE. 2020. Species identification and antibiotic resistance prediction by analysis of whole-genome sequence data by use of ARESdb: an analysis of isolates from the Unyvero lower respiratory tract infection trial. J Clin Microbiol 58:e00273-20. doi:10.1128/JCM.00273-20. 32295890 PMC7315026

[38] Clinical Laboratory Standards Institute. 2015. M52 Verification of Commercial Microbial Identification and Antimicrobial Susceptibility Testing Systems. 1st ed. Clinical Laboratory Standards Institute, Wayne, PA.

